# Exploring food poverty experiences in the German Twitter-Sphere

**DOI:** 10.1186/s12889-024-18926-8

**Published:** 2024-05-25

**Authors:** Tina Bartelmeß, Mirco Schönfeld, Jürgen Pfeffer

**Affiliations:** 1 Faculty of Life Sciences: Food, Nutrition and Health, Professorship of Food Sociology, University of Bayreuth, Fritz-Hornschuch-Strasse 13, Kulmbach, Germany; 2 Faculty of Languages and Literature, Professorship of Data Modelling and Interdisciplinary Knowledge Generation, University of Bayreuth, Nürnberger Straße 38, Bayreuth, Germany; 3 Munich School of Politics and Public Policy, Professorship of Computational Social Science and Big Data, Technical University of Munich, Arcisstraße 21, Munich, Germany

**Keywords:** Food insecurity, Food poverty, Germany, Hashtag, Multidimensional deprivation, Twitter

## Abstract

**Background:**

This study investigates the subjective perceptions of food poverty in Germany by analysing Twitter discourse using the German-language hashtag #IchBinArmutsbetroffen (#IamPovertyAffected) and examines the extent to which various dimensions of a multidimensional theoretical model of food poverty are represented in the discourse.

**Methods:**

Employing a combination of computational social science and qualitative social research methods, the research identifies, and analyses tweets related to nutrition by applying a hierarchical dictionary search and qualitative content analysis. By examining the narratives and statements of individuals affected by food poverty, the study also investigates the interplay among different subdimensions of this phenomenon.

**Results:**

The analysis of 1,112 tweets revealed that 57.96% focused on the material dimension and 42.04% on the social dimension of food poverty, suggesting a relatively balanced emphasis on material and social aspects of food poverty in the narratives of those affected. The findings reveal that tweets on material food poverty underscore economic challenges and resource scarcity for food. Social food poverty tweets demonstrate widespread deprivation in social participation, leading to isolation, exclusion, and social network loss. Overall, the results elucidate intricate interconnections among subdimensions and multidimensional manifestations of food poverty.

**Conclusions:**

This study contributes methodologically by presenting an approach for extracting food-related textual social media data and empirically by providing novel insights into the perceptions and multifaceted manifestations of food poverty in Germany. The results can aid in a better understanding of the phenomenon of food poverty as it currently manifests in Germany, and in developing targeted social, health-promoting, and political measures that address more effectively the empirically evident multidimensionality of the phenomenon.

**Supplementary Information:**

The online version contains supplementary material available at 10.1186/s12889-024-18926-8.

## Introduction

Food poverty has witnessed a rise in industrialized nations, including Germany, notably precipitated by the crises instigated by the COVID-19 pandemic, the Russian war of aggression on Ukraine, and the resultant inflationary pressures [1–4]. Affected individuals have availed social media platforms, such as Twitter, as channels to articulate their experiences and concerns. This led to the German-language hashtag #IchBinArmutsbetroffen (English: #IamPovertyAffected) emerging on the social network Twitter on May 12, 2022, initiated by a single mother using the pseudonym ‘Finkulasa’^1^. This hashtag brought public attention to her experiences of poverty, with a specific focus on the challenges related to nutrition in impoverished circumstances. Subsequently, many individuals affected by poverty adopted this hashtag to share their experiences through tweets. These tweets offer valuable insights into the daily lives of people living in poverty, shedding light on how poverty impacts their nutrition, health, and overall social well-being.

As of 2022, approximately 21% of the German population were at risk of poverty or social exclusion [4]. Various factors, including the COVID-19 pandemic, rising energy and food prices resulting from the Russian invasion of Ukraine, supply chain disruptions, and subsequent increases in food prices and inflation, may have exacerbated the situation [5–7]. These significant price increases primarily affect low-income and poverty-vulnerable demographic groups and resulting financial constraints have immediate repercussions on dietary patterns and health as nutrition research consistently highlights [8]. But even households with income levels exceeding 60% of the median equivalized income faced material deprivation in terms of nutrition at a rate of nearly 10% in 2022 [3]. As a result, in 2022, according to the most recent data from EU-SILC, 11.4% of the German population experienced material deprivation in terms of nutrition [3]. This equates to an estimated 9.6 million individuals in Germany who could not access a balanced meal consisting of meat, fish, or a vegetarian equivalent on alternate days.

These numbers solely encapsulate the statistical dimension of empirical food poverty measurement and its implications for nutritional and health-related outcomes. They oversimplify the multifaceted nature of food poverty, which fundamentally encompasses a daily existence characterized by constraints, deprivation, and social exclusion [9, 10]. The definition and measurement of poverty and material food deprivation are primarily based on financial resources and constraints resulting from financial shortages. Certainly, nutrition unquestionably serves as a crucial indicator of one’s quality of life, manifesting material deprivation at an early stage [10]. However, the predominant emphasis on financial resources as the principal determinant for measuring food poverty presents a limited, one-dimensional perspective. Nutrition’s significance extends to socio-cultural participation, emotional well-being, and individual health [11, 12]. Food serves purposes beyond mere sustenance, satisfying emotional, social, and cultural needs that impact on identity and social and family life [13–15]. Nonetheless, there exists a significant deficiency in understanding the multifaceted aspects of food poverty and its subjective perception within the context of German society.

A recent report by the Scientific Advisory Board for Agricultural Policy, Nutrition, and Consumer Health Protection (WBAE) has underscored the urgency of systematically investigating food poverty and implementing appropriate measures for its alleviation [16]. However, while research and policy efforts are gradually turning their attention to this topic, individuals affected by poverty in Germany in the middle of 2022 have found a way to make their voices heard through the social media platform Twitter. The emergence of the above-mentioned hashtag was catalysed by the political debates surrounding the replacement of the former state-provided social welfare program for securing the minimum subsistence level and the consequent renegotiation of standard rates for daily living expenses. This was coupled with the media’s stigmatizing and stereotypical portrayal of those affected by poverty, who are frequently accused, particularly from political circles, of being incapable of managing their finances [17, 18]. Using the German-language hashtag #IchBinArmutsbetroffen, Twitter users have been sharing tweets to ascertain the true identities and lived experiences of individuals residing in poverty within their daily existence, while also advancing collective political demands.

This study takes this hashtag-based discourse as an opportunity to explore how food poverty in Germany is subjectively perceived by those who experience it. The study employs methods from computational social science and qualitative social research to identify and analyse nutrition-related tweets with the hashtag #IchBinArmutsbetroffen. Based on a theoretical multidimensional model of food poverty [19], the tweets are analysed to examine the extent to which individual dimensions are represented. The objective is to reveal manifestations of food poverty that surpass findings obtained from ongoing poverty and health statistics in Germany, providing deeper insights into the multidimensional deprivations experienced by those affected in their daily lives. Based on the statements and narratives of those affected about their experience of food poverty, the study also examines how different subdimensions of food poverty are interrelated. Consequently, the study makes a twofold contribution: methodologically, it presents an approach for identifying and extracting food-related textual social media data, while empirically delivering novel insights into the perceptions and multidimensional manifestations of food poverty in Germany. Highlighting the prevalence of individual dimensions, their interdependence, and the multidimensionality of the food poverty phenomenon as it currently manifests in Germany can aid in a better understanding. Leveraging this understanding can facilitate the development of targeted social, health-promoting, and political interventions that more effectively address the empirically observed multidimensional nature of the phenomenon. This, consequently, can aid in alleviating the stigmatization and bias faced by individuals experiencing food poverty within public and political discourses.

The remainder of the paper is structured as follows: the next section introduces the concept of food poverty and its individual dimensions. Emphasis is placed on elucidating the significance of social media utilization as well as its potential as a voice amplification tool for vulnerable groups. Following this, we delineate the methodological design, present the results, and conclude with a comprehensive discussion encompassing the results, methodology, and derived limitations, along with implications.

## Food poverty dimensions and representations

The definition of food poverty is marked by a lack of consensus, a situation that poses significant challenges for both academic and political examinations. This discord hampers efforts to quantify the number of individuals falling under this classification and impedes the formulation of effective strategies to address this issue [9]. Feichtinger [19], drawing upon Townsend’s theory of deprivation [20], has developed a comprehensive framework for understanding food poverty. This framework extends beyond material dimensions to incorporate social aspects, thereby shedding light on the psychological, social, and cultural consequences of poverty experiences. According to Feichtinger [19], material food poverty pertains to a diet that is “inadequate in terms of quantity and lacking in physiological and hygienic quality” [19]. Material food poverty is subdivided into four subdimensions. The economic dimension pertains to the lack of financial means for accessing food. The physical dimension relates to spatial access to food or provisioning facilities. The physiological dimension is concerned with inadequate nutrient and energy intake, as well as low nutritional quality of food. Lastly, the hygienic dimension pertains to the availability of hygienically safe and secure food.

In contrast, social food poverty accentuates the intangible social, cultural, and psychological dimensions of nutrition as indicators of deprivation. It places emphasis on various societal aspects of nutrition as fundamental components of social and cultural participation and psychological well-being. Social food poverty refers to a diet “that does not permit the establishment of socially accepted relationships, the assumption of roles and functions, the exercise of rights and responsibilities, or the observance of customs and traditions associated with the social and cultural aspects of food within a society” [19]. It comprises a social subdimension that refers to social organization, integration, and delineation through food and nutrition. A cultural subdimension that alludes to normative value systems, dietary customs, and traditions. And a psychological subdimension that points to aspects such as pleasure, emotional security, compensation, and self-esteem in relation to food and eating. However, social food poverty and its subjective experiences and consequences remain relatively underexplored, primarily due to the inherent challenges in reaching and representing vulnerable groups in comprehensive studies and scholarly research [21]. Investigating food poverty at the household or individual level poses additional difficulties, as individuals may harbour feelings of embarrassment when disclosing information, they perceive as shameful [22].

A review of the limited research in this field reveals that food poverty profoundly permeates individuals’ lives, exerting substantial influence on their daily routines within domestic, educational, and occupational contexts. Some research has indicated that individuals, particularly mothers, occasionally forego meals to ensure their children have enough to eat [23, 24].

Qualitative studies conducted to date illustrate the intricate and multifaceted nature of food poverty. Notably, food poverty not only affects food availability and quality of diets, thereby influencing health outcomes but also impedes social engagement related to food [22]. Food serves as a medium for both social exclusion and inclusion [25]. Low-income households often face challenges when attempting to host friends for meals [23, 24, 26]. Food poverty exerts a pervasive impact on individuals’ social relationships, influencing interactions with immediate family, friends, and neighbours, a phenomenon referred to as ‘alimentary participation’ [11]. Additionally, food poverty plays a pivotal role in shaping individuals’ decision-making processes and strategies for food procurement, all while inflicting emotional distress due to a persistent fear of food scarcity [22].

Recognizing the significant role played by the media in shaping policy discourses and public attitudes toward poverty, qualitative investigations have revealed that media coverage predominantly focuses on individuals utilizing food banks when portraying food poverty [27]. While empirical investigations have also delved into the experiences encountered by individuals utilizing food banks, providing insights into the feelings of embarrassment and stigma associated with such reliance [18, 28], studies examining media discourse have illuminated that public media frequently mirrors a predominant discourse and ideology that attributes blame to individuals residing in poverty. These portrayals often label them as ‘scroungers’ and ‘spongers’ who depend on state support, characterizing them as ‘frauds’ displaying an unwillingness to engage in employment and having made ‘erroneous choices’ [29–31]. These discourses are argued to perpetuate a mythical narrative linking family dysfunction, unemployment, and welfare dependence [32], furthering the process of ‘othering’ people living in poverty, wherein those living in poverty are marginalized and devalued, often manifesting through a lack of representation and acknowledgment in public discourses [33]. These discursive practices tend to emphasize individual shortcomings, diverting attention away from the underlying structural social inequalities that contribute to poverty. Hence, to provide the public with a more accurate portrayal of the experiences of food poverty, it is imperative to delve deeper into its multifaceted nature. Following 27 [27], this includes recognising food as a basic human right, acknowledging the social dimensions of food and empowering the voices and experiences of those affected. However, if these aspects are not integrated into the prevailing public and political discourses, media coverage is unlikely, consequently impeding the broader public’s awareness of these issues [27].

## Social media as a voice amplifier for food poverty experiences

Social media platforms such as Twitter, Instagram, TikTok and Facebook are characterized by low-threshold social access, as they fundamentally enable any internet user to make information publicly accessible. They primarily differ in terms of their specific content formats and enjoy varying popularity among different age groups [34]. Twitter, for instance, is a microblogging platform designed for the dissemination of short messages in text and image format. It is globally typically utilized by individuals aged 25 and above [35].

In the realm of social media platforms, hashtags have emerged as powerful tools for giving voice to marginalized groups in society and shedding light on personal experiences that might otherwise remain hidden in the absence of social media [36–39]. Hashtags enable content categorization and provide users with the means to track conversations related to specific subjects, offering insights into discussions on a given topic that they might not otherwise encounter [40]. Hashtags serve as “multimodal discourse markers” [41], conveying not only information within a tweet but also integrating those tweets into broader discussions on the same subject. Furthermore, hashtags have evolved into a formal tool for raising awareness and driving social change. By using hashtags, individuals can draw attention to issues of societal relevance and highlight information that might otherwise remain overlooked in mainstream media narratives [38]. Hashtag utilization on social media platforms enables “networked gatekeeping” [42] allowing individuals to gain visibility by collaboratively shaping information within hybrid and dynamic information flows, creating ‘affective’ news streams characterized by a blend of emotions, opinions, personalized information sharing, and self-disclosure [42, 43]. Moreover, the use of hashtags promotes solidarity, a critical element of social movements [44], fostering connections among like-minded users [41, 45].

The analysis of discourses surrounding hashtags on Twitter has been applied across various disciplines and for diverse purposes, such as sustainability discourses [46, 47], political discourses [48, 49] or health and nutrition discourses [45, 50, 51]. These studies have demonstrated that Twitter data are valuable for gaining insights into perspectives that are often challenging to access and underrepresented in empirical social research. Furthermore, they have shown that Twitter data provide real-time datasets containing reactions, attitudes, and opinions regarding current events. Although Twitter data have been increasingly recognized as valuable sources of geographical metadata, behavioural data, visual content, and linguistic data that offer insights into users’ daily lives, opinions, emotions, and behaviours, research endeavours focusing on food poverty or food insecurity using Twitter data have been relatively scarce. The terms “food poverty” and “food insecurity” are frequently employed interchangeably [52]. Investigations into food insecurity typically prioritize considerations of food availability, affordability, and accessibility. In contrast, examinations of poverty commonly acknowledge that the capacity to procure an adequate food supply is fundamental, yet concurrently underscore its association with or influence on the ability to satisfy nutritional requirements and conform to social norms in dietary behaviour [53]. The work on both terms is taken into account here, but in the further course of the data analysis reference is made exclusively to the theoretical assumptions on food poverty [10, 19]. Some social media-related studies on food insecurity or poverty have analysed food-related tweets and metadata to identify geographical dynamics or food deserts associated with inadequate access to healthy foods [54, 55]. Other studies have focused on sentiment analysis to examine emotions or reactions expressed on Twitter, particularly in response to emergency food supplies [56]. From a discourse analytical perspective, some studies have explored the thematic complex of food insecurity and food poverty, investigating the framing of hunger in public discourse and its use as a political tool to assert moral claims on the state [57]. However, there is only one study to date that directly addresses food poverty, with a primary emphasis on methodological approaches rather than providing empirical evidence within a theoretical framework for food poverty. Eskandari et al. [2] conducted a quantitative network analysis of a corpus of 81,249 tweets containing the terms ‘food’ and ‘poverty’ in the year 2020. Their data collection coincided with the global lockdowns and contact restrictions imposed during the COVID-19 pandemic, highlighting how food-secure families rapidly transitioned into food insecurity due to surging unemployment and increasing poverty rates resulting from quarantine measures and stay-at-home directives.

Despite the advantages and data offered by social media and hashtag analysis, it is important to note that, to date, profiling users in terms of their socioeconomic and demographic characteristics has been challenging. The specific socioeconomic characteristics of users participating in the discourse under study—such as income, education, and occupation—are often not conclusively available in social media data.

## Methods

### Data collection

The data collection process is two-stage, as first all tweets with the hashtag #IchBinArmutsbetroffen were collected, and then food-related tweets were identified from the total of all tweets. The initial data for this study come from a dataset of 74,832 original tweets and retweets containing the hashtag. The tweets were collected in November 2022 using the academic Application Programming Interface (API) of Twitter [58]. Data collection was restricted to publicly available tweets. The sample spans 180 days of activity, from 12 May 2022 to 8 November 2022. This period covers discussions from the initial launch of the hashtag (12 May 2022) and the course of discussions over the following 26 weeks. This timeframe was chosen because the discussions under the hashtag were highly intensive during this initial period on Twitter, while at the end of 2022 additional platforms such as Instagram as well as the hashtag’s own website were established, and the discussions thus shifted to other platforms. However, following the approach proposed by Pfeffer et al. [58], we ensured that our sample included all tweets that were posted within the analysis period and contained the relevant hashtag.

Hence, our data set contained a total of 7,734 unique users of the hashtag were identified, indicating the reach of the hashtag. The frequency of hashtag usage in the collected data period varies greatly with peaks along the entire period and decreasing frequency of usage from the beginning to the end of the period, with, for example, a peak of 1,397 tweets with the hashtag per day on 31 May 2022 to a drop of 129 tweets with the hashtag on 28 October 2022.

To identify the food-related tweets, lexicons were created. As the discussions under the hashtag #IchBinArmutsbetroffen are German-language, these lexicons were also created based on German-language terms taken from dietary guidelines of the German Nutrition Society, the co-occurrence database of the Leibniz Institute for the German Language and other dictionaries. Overall, the lexicon comprises approximately 750 terms. The lexicon categories were clustered into three larger areas covering the domains eating, diet and food. The area of eating comprises eight subcategories consisting of terms that refer, for example, to the activities of eating, to the preparation or also to dishes. The diet domain includes terms that refer to diets, nutrients, health aspects and physiological processes related to nutrition. It also includes eight subcategories. The category of food includes 17 subcategories, which on the one hand encompass terms related to shopping, supermarkets, brands, and qualities, and on the other hand, list specific foods based on food groups.

**Fig. 1:**
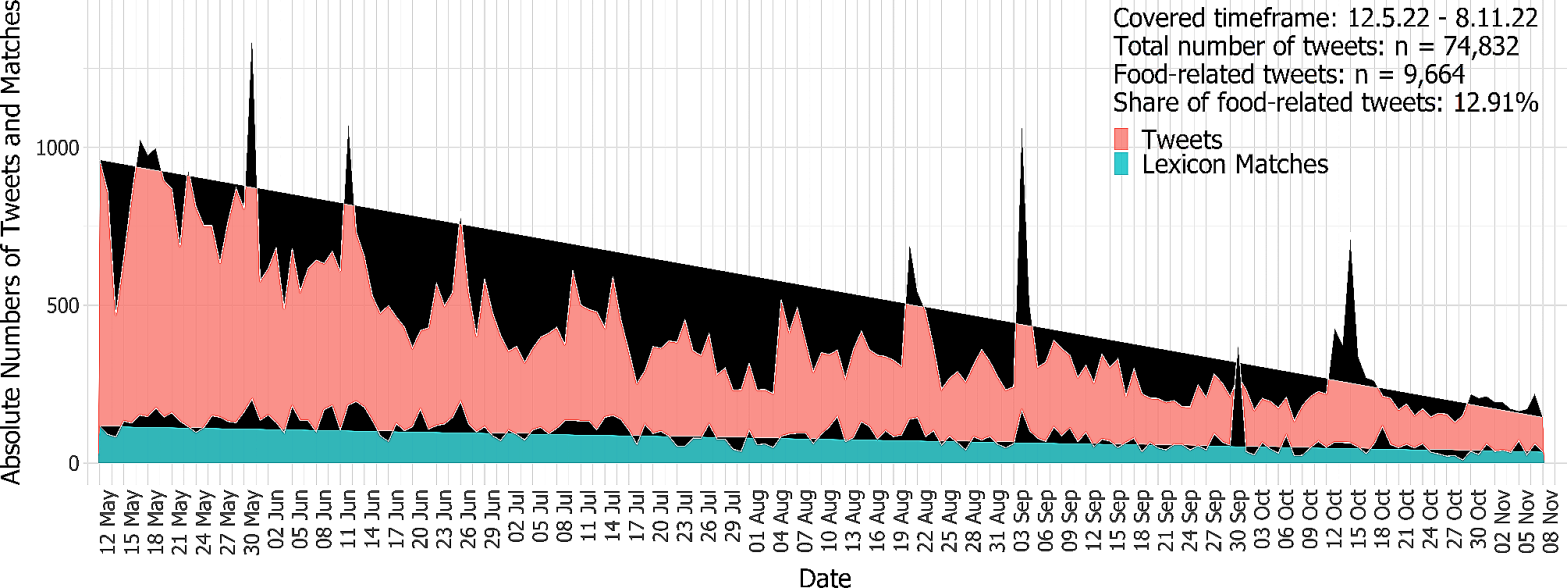
Tweets and share of food-related tweets over time

This hierarchical dictionary was then used to perform a lookup on the entire Twitter data set using R and the text analysis library quanteda. The results were made available as an interactive web-app based on shiny (#ichbinarmutsbetroffen (uni-bayreuth.de)). Subsequently, the generated food-related data corpus was reviewed, and false positives, erroneously identified by the lexicon entries, were manually filtered out. The final food-related corpus comprises 9,664 tweets, constituting a 12.91% share of the total tweets under the hashtag #IchBinArmutsbetroffen (see Fig. 1).

The lexicon category that identified the most food-related tweets is that of activities related to eating (E_TAETIG, see Table 1). This category identified 2,820 tweets as food-related and includes terms that refer to general activities related to food. Other lexicon categories that yielded many hits were the category of food consumption with general terms indicating the consumption of food, identifying 1,245 tweets (LMK_B, see Table 1), as well as the category of the food group encompassing non-alcoholic beverages (LMG_G) with 1,296 hits.

**Table 1:** The 10 most matching lexicon categories in the food-related data corpus

Rank	Lexicon category	Frequency	Description	Exemplary terms
1	E_TAETIG [eating activities]	2820	Terms that indicate activities of eating	*ERNAEHR*; ESSE*; FASTE*; NASCH*; SCHLEMM*
2	LMK_B [food consumption terms]	1545	General terms that indicate the consumption of food or discussions centred around food in general	*FOOD*; *KONSERV*; *KOST; *LEBENSMITTEL*; *NAHRUNG*
3	LMG_G [food groups beverages]	1296	Terms that refer to the group of non-alcoholic beverages	KAFFEE; *LATTE; *SAFT; *TEE; SPRUDEL*
4	ERN_PHYS [diet physiology]	1068	Terms that indicate physiological processes related to nutrition	APPETIT*; DURST*; *HUNGER*; KNURREN; SATT
5	LMK_HANDEL [food consumption supply]	913	Terms that indicate shopping locations or other food supply establishments	ALDI; BAECKER*; *DISCOUNT*; LIDL; *SUPERMARKT; TAFEL
6	LMG_GEM [food group vegetables]	884	Terms that refer to the food group vegetables	BROKKOLI*; CHAMPIGNON*; GEMUESE*; KARTOFFEL*; SPARGEL
7	EVERS_TAETIG [food provisioning activities]	855	Terms that indicate to activities of food provisioning	BEWIRT*; EINKAUF*; VERPFLEG*; *VORRAT*; VORRAET*
8	EZUB_TAET [food preparation activities]	688	Terms that indicate activities of food preparation	*BACK*; *BRATE*; EINMACH*; *KOCH*; PUERIER*
9	EZUB_GERAET [food preparation devices]	682	Terms that indicate devices for eating or food preparation	*BLECH; *FRITEUSE; LOEFFEL; *MIXER; *TOPF
10	E_GER [eating dishes]	641	Terms that refer to dishes or meals	*AUFLAUF; *KETCHUP; *PESTO; *POMMES; *SUPPE

It is important to acknowledge that within the tweets, there exists an intersection among the matching categories. Consequently, the focus is not on individual identified tweets but rather on the cumulative count of tweets identified by each specific lexicon category.

### Data analysis

To address our research question, we limited the corpus to all tweets identified through the lexicon category ‘eating activities’ (E_TAETIG), which constituted a total of 2,820 tweets. This subset represents tweets over the entire data period, the determinant lexicon category is the one that most frequently overlaps with the other lexicon categories used and is thus thematically the most representative for the overall corpus and it is manageable in size [59]. The subset data were imported into a project file and coded using MAXQDA Analytics Pro 2022 software for qualitative analysis. We used qualitative content analysis to systematically quantify and synthesize the content in terms of predetermined categories [60]. We deductively derived a coding frame based on the theoretical discussions of food poverty dimensions [19]. Deductive qualitative analysis allows emerging themes to be linked to theory so that the insights gained can fill gaps in current understanding of a particular theme [61], such as patterns and characteristics of the dimensions of material and social food poverty. The initial categories included the material (economic, physiological, physical, and hygienic) and social (social, cultural, and psychological) subdimensions of food poverty [8, 19]. In addition, we coded the data inductively based on the themes that emerged from the tweets, which resulted in a refinement of the deductive codes and additional main codes. Coding allowed for tweets to be categorized without exclusivity, meaning that they could be assigned to multiple categories simultaneously.

In our data analysis, our primary focus was on original tweets, excluding retweets, as well as tweets identified by the lexicon category but subsequently deemed content irrelevant. This exclusion was carried out to mitigate the potential bias introduced by the frequent recurrence of identical linguistic descriptions in retweets or irrelevant statements not related to the research subject. Consequently, the subsequent analysis is predicated upon a total of 1,982 original tweets. Subsequently, 838 tweets were manually filtered out of the category ‘eating activities’ in the qualitative analysis. After the first researcher was through with about half of the material, to assess reliability, a total of 480 tweets (25% of the total tweets included) were coded by a second researcher. The intercoder reliability coefficient was computed using MaxQDA, yielding a Kappa according to Brennan et al. [62] of 0.81 for the selected six primary categories with sub-categories, indicating excellent agreement. Coding discrepancies were discussed, and the definition of coding criteria was improved to eliminate inconsistencies and make the further coding process more robust. Adhering to ethical principles in web research, the tweet featured in this paper was translated into English, references to individuals and names were removed and the tweets in the supplementary material were mildly paraphrased to safeguard the anonymity of the Twitter users [63].

During the coding process, it was possible to assign the same tweets to multiple codes, enabling the examination of significant overlaps between individual subdimensions of food poverty in the perceptions of Twitter users. To elucidate the intertwined nature and multidimensional manifestations of food poverty, the overlaps of codes in the respective tweets were computed using the Code-Relations-Browser (CRB) tool from MAXQDA 2022, resulting in a matrix. Subsequently, this matrix was visualized on a map to demonstrate the extent to which codes overlap in the tweets and are consequently associated with each other (see supplementary material, Table S2, Figures S1 and S2). To position the codes on the map MAXQDA 2022 uses classical multidimensional scaling (MDS) technique. An initial similarity matrix is computed based on the representation found in the CRB. This similarity matrix is then transformed into a distance matrix, upon which a cluster analysis is conducted. This analysis aimed to illustrate the degree of similarity in the utilization of codes within the dataset and consequently, how the individual subdimensions of food poverty are interconnected.

## Results

In the sub-dataset consisting of 1,982 original tweets related to food with the hashtag #IchBinArmutsbetroffen, we conducted 1,920 codings to capture the dimensions of food poverty as expressed in the tweets. Table 2 provides a quantitative overview of the subdimensions of food poverty most frequently addressed in the tweets.

**Table 2:** Number of codes and percentages per food poverty dimension

Subdimension	Number of codings	Share on total codings
Material food poverty		
economical	637	33,18%
physiological	459	23,91%
physical	16	0,83%
hygienic	0	0,00%
*sub-total*	*1,112*	*57,96%*
Social food poverty		
social	326	16,98%
cultural	93	4,84%
psychological	389	20,26%
*sub-total*	*808*	*42,04%*
total	**1920**	**100,00%**

Most tweets, comprising 1,112 codings and accounting for 57.96%, were assigned to the material dimension of food poverty. Within this category, 33.18% pertained to the economic subdimension, 23.91% to the physiological subdimension, and 0.83% to the physical subdimension. No tweets were identified directly addressing the hygienic subdimension, indicating limited reporting on restricted access to hygienically safe food.

Regarding the social dimension of food poverty, a total of 808 codings were assigned, constituting 42.04% of all codings. Tweets most frequently discussed deprivations in the psychological dimension, accounting for 20.26% of all codings, followed by 16.98% of codings related to the social subdimension of food poverty. The cultural subdimension was minimally addressed in the tweets, representing only 4.84% of all codings. The ratio of codings between the material and social dimensions of food poverty suggests that while the material aspect is somewhat more frequently discussed overall, there is a relatively balanced emphasis on these two dimensions of food poverty, highlighting their roughly equal importance to those affected.

The following section presents the results organized by each dimension of food poverty and subsequently highlights significant overlaps among the various dimensions of food poverty in the perceptions of the Twitter hashtag users, thus showcasing the multidimensionality of deprivation due to food poverty.

### Material food poverty

#### Economic subdimension

The most prevalent aspect of material food poverty discussed in tweets, accounting for 33.18%, pertains to the economic dimension, signifying a lack of financial resources to acquire food. Seven subcategories emerged within the dataset, reflecting people’s thoughts and perceptions.

The most discussed subcategory, with 204 codings, involves calls for government action or criticism of politics. This encompasses demands for government intervention in addressing food poverty, criticism of politicians for their inaction, and frustration regarding inadequate government support for healthy food options. Related discussions encompass basic income, standard allowances, and social security (131 codings), as well as price increases and inflation (86 codings), highlighting concerns about the inadequacy of proposed government assistance considering rising costs. These tweets underscore the necessity for systemic reforms to combat food poverty and ensure equitable access to affordable, nutritious food. Suggestions, such as eliminating VAT on fruits and vegetables and reducing the cost of living, are proposed as potential solutions. The role of food banks, known as ‘Die Tafel’ in Germany, is also discussed, with many relying on them due to financial constraints. However, concerns are raised about the quality and quantity of food provided by food banks and their sustainability as a solution to manage food poverty in Germany. Additionally, individuals with disabilities or chronic illnesses encounter difficulties accessing food banks, exacerbating the challenges faced by those in poverty. Some argue that it is the government’s responsibility to ensure universal access to nutritious food, making food banks redundant.

Another subset of tweets discusses how financial constraints impact various aspects of food provision and daily life. A few tweets (8 codings) highlight inadequate household equipment, such as ovens or refrigerators, due to financial limitations, affecting food preparation and storage. There is also discussion (40 codings) about the ‘mid-month’ phenomenon, where people are already accumulating debt around the 15th of the month under current circumstances, leaving no money for food. Moreover, there is significant discussion (129 codings) about how financial restrictions force individuals to choose between food and other essentials, such as electricity, gas, or transportation. Many must prioritize spending, often sacrificing items like new clothing or entertainment to afford food. Medical expenses also pose challenges, with some individuals having to choose between necessary medication and purchasing food, a consideration that appears as a heuristic influencing all economic decisions of those experiencing food poverty.

#### Physiological subdimension

The physiological dimension, accounting for 23.91% of total codings (459 codings), is a significant discourse area in the tweets. Within this dimension, four subcategories have emerged. The most prevalent subcategory is strategies for achieving appropriate nutrition with limited financial resources (185 codings). Many individuals report sacrificing their own meals for other family members and, when they do eat, consuming leftovers. Eating is not driven by hunger or preferences, but by strategies aimed at minimizing hunger. Some individuals experience hunger due to involuntary fasting, reducing food quantity and meal frequency, and sometimes substituting with vitamin tablets. Food is stretched, meals are planned meticulously, and special offers are sought. Food wastage is avoided, even if it means consuming spoiled food. Furthermore, the feasibility of healthy eating in poverty is extensively discussed and commented (140 codings). Impoverished individuals find it challenging to access nutritious and sustainable food options, including vegan, vegetarian, organic, or locally sourced choices. The cost of animal-derived products and highly processed foods is often below that of healthier alternatives, making it difficult for those with limited resources to make healthier dietary choices.

Moreover, statements from impoverished individuals with specific dietary needs and health challenges (81 codings) emerge. Those dealing with diseases, intolerances, or allergies mention the financial burden of specialized diets. Inadequate nutrition can weaken the immune system, leading to recurrent infections and increased medication costs. Many individuals cannot afford necessary specialized diets, medications, or medical treatments due to financial constraints, exacerbating their health issues and potentially reducing life expectancy. Poverty generally leads to difficulties in affording nutritious food, resulting in suboptimal nutrition and potential health problems, including vitamin and micronutrient deficiencies. Malnutrition is directly addressed in the tweets (53 codings), with individuals attributing it to their dietary practices in poverty and feeling helpless due to financial constraints. Malnutrition perpetuates poverty, creating a challenging cycle to break.

#### Physical subdimension

The physical subdimension of food poverty is only minimally addressed in the tweets (16 codings). Topics discussed include the lack of food supply from food banks in rural areas and the appreciation expressed by affected individuals when they have their own garden or space for growing food to sustain themselves. Additionally, we have coded statements related to altered or extreme climatic conditions in the physical environment within this dimension, as some tweets occasionally mention concerns and potential health consequences associated with extreme heat, for example. A concern that may not be entirely noticeable and conscious to many in their geographical surroundings today but is likely to increase in the future.

### Social food poverty

#### Social subdimension

The social subdimension of social food poverty represents 16.98% of all codings (326 codings). Within this subdimension, four subcodes were established, with most tweets categorized under ‘social participation’ (154 codings). These tweets describe how individuals experiencing poverty often cannot afford to socialize by going out for drinks or dining in cafeterias or restaurants with family, friends, or colleagues. Consequently, social isolation and exclusion are prevalent experiences for those in poverty, as they lack the financial means for social activities that others take for granted. Chronic illness can further exacerbate social isolation and limit access to such activities. Additionally, sacrificing hobbies and interests due to financial constraints is a common occurrence. These tweets emphasize that affected individuals are less concerned about dining or consuming beverages outside their homes and more interested in the social aspects associated with communal meals and conversations in public places, such as the enjoyment of life and the sense of community fostered by shared meals and conversations over food and drinks.

Another significant category is ‘social networks’ (80 codings). Here, individuals describe how limited social participation leads to the decrease of social networks and the loss of friendships. Moreover, inviting people into their homes can necessitate difficult choices and careful budgeting for additional meals when hosting guests. Many accounts revolve around children, who may be affected by limited opportunities for socializing and inviting friends over. A significant number of individuals rely on the generosity of friends and family to attend events and access fresh food. However, possessing a support system of friends and family who can assist with expenses is a privilege not enjoyed by everyone facing poverty. Those who can rely on a social network tend to identify themselves on Twitter as younger individuals, including students. For individuals in middle and older age groups, charitable organizations operating on Twitter under hashtags such as #bratenpaten (roast sponsors), #einesorgeweniger (one worry less), or #wishlistengel (wish list angel) appear to be significant. These organizations enable anonymous donors to fulfil the wish lists of those in need (such as items from an Amazon wish list) or provide food vouchers funded by donations to affected individuals.

Additionally, a notable portion within this subdimension discusses the perception of ‘social roles’ affected by food and nutrition, particularly within the parental role. The most common scenario described is parents sacrificing their own meals and needs to prioritize the health and nutrition of their children. Some individuals recount challenges in affording school meals or extracurricular activities involving food, resulting in children going without meals and the associated fear of parents being reported to social services due to their inability to provide food. Parents also express feelings of guilt for not being able to provide specific foods or experiences for their children. They also report experiencing shame when their mostly adult children take on expenses for dining out or similar activities during celebratory occasions, such as a university graduation, which, according to societal conventions, should typically be covered by the parents. A smaller but notable portion of the tweets also addresses the influence of food poverty on ‘social imprint’ (19 codings). These tweets describe how experiencing poverty from a young age can lead to specific habits and behaviours that persist even when no longer required. Children may become aware of and influenced by their family’s financial challenges, such as the inability to dine out or the necessity to sell toys to procure essential items like food. Parents recount how their children develop an appreciation for symbolically valuable food at a young age and how certain frugal behaviours towards food become ingrained.

#### Cultural subdimension

The cultural subdimension underscores deprivations within normative food value systems, dietary customs, and practices, representing 4.84% of all codings (93 codings) within a less frequently discussed domain of deprivation. This subdimension comprises two primary subcategories, with ‘symbolic food and practices’ being the most prominent (73 codings). It reveals that certain foods and beverages continue to symbolize status distinctions for individuals affected by poverty, although their access to such items is limited. These items include primarily healthy foods like watermelon or strawberries, items representing sociocultural trends (e.g., sushi), seasonal practices (e.g., eating ice cream), as well as foods like steak and alcoholic beverages associated with social participation. Conversely, items like toast, rice, potatoes, or pasta are perceived as indicators of poverty. Some individuals experience guilt or anxiety when consuming specific foods due to financial constraints. Nevertheless, individuals facing poverty find moments of joy in small indulgences, such as preparing dishes that evoke pleasant memories or participating in cultural food practices, such as using a muffin baking tray.

Another aspect of cultural food poverty pertains to ‘festive occasions’ (20 codings). This dimension highlights the impact of food poverty on significant life events like weddings, birthdays, traditional festivities such as Christmas or Easter, and public celebrations. Individuals often struggle to contribute financially or gather the necessary means to prepare customary dishes for these events. Some mention forgoing meals to save money for important occasions or expenses. Additionally, the dataset reveals that people in poverty frequently experience anxiety and fear regarding events like birthdays or Christmas due to the associated expenses, including gifts and food. While some have had the opportunity to enjoy traditional Christmas meals thanks to organizations like #Bratenpaten (roast sponsors), many families face the difficult choice between providing a warm meal or purchasing gifts for their children due to the rising cost of living.

#### Psychological subdimension

In the analysed tweets, social food poverty emerged as the predominant theme, constituting 20.26% of all codings (389 codings). This dimension primarily pertains to the psychological aspects of food poverty, encompassing five subcodes. Notably, ‘dignity and related justifications’ was the most prevalent subcode, with 178 codings. These tweets underscore the courageous sharing of experiences by individuals experiencing poverty on Twitter, emphasizing the importance of avoiding shame and blame. They often face judgment and criticism, both on Twitter and in daily life, highlighting disparities in treatment compared to more affluent individuals. Politicians and those in positions of authority are often criticized for making dismissive and uninformed comments about poverty. Simplistic advice on affordable and nutritious food choices or austerity measures, along with impractical and derogatory suggestions for saving money on food, are deemed unhelpful and disrespectful to the dignity of those in poverty. Criticisms of food choices and discussions about food literacy prevail, even when individuals are striving to maintain a healthy diet on a limited budget. People facing poverty often feel compelled to justify modest indulgences like bottled water or flavoured beverages, advocating for the principle that low-income individuals have the right to dignity and access to a nutritious diet without enduring criticism for their choices.

Additionally, the tweets report a wide range of emotions, thoughts, and fears (88 codings) experienced by individuals facing food poverty. These include feelings of shame, isolation, and anxiety. Concerns about inadequate food availability can lead to existential fears, persistent hunger, and an obsessive focus on food. Rising food prices and financial instability exacerbate these emotions, contributing to feelings of isolation and despair. People in food poverty express a sense of neglect and being overlooked by politicians, who they perceive as inadequately addressing poverty and food insecurity. References to shame and associated societal prejudices are also prevalent in the tweets (70 codings). They highlight how poverty often triggers feelings of shame and fosters prejudicial attitudes, including false assumptions about resource allocation toward cigarettes, alcohol, or drugs. Regarding nutrition, tweets emphasize the misconception that impoverished individuals exclusively consume convenience or unhealthy foods, neglecting the challenging choices they face in allocating limited resources between nourishing food and essential expenses. Stigmatization and stereotypes, such as laziness and irresponsibility, place undue pressure on individuals affected by poverty, despite many being diligent and hardworking individuals struggling to make ends meet. Those affected emphasize that the lack of understanding and empathy toward poverty often leads to the neglect of systemic factors contributing to the issue.

Furthermore, stress and deprivations are only minimally addressed in the tweets (28 codings). Depression and anxiety frequently manifest among individuals living in poverty, with financial insecurity exacerbating these conditions. The scarcity of resources and ongoing financial concerns create a constant state of stress and fatigue. Additionally, social isolation resulting from financial constraints and the inability to participate in activities further worsens mental well-being. Discussions of unconventional coping strategies are relatively infrequent (25 codings). These tweets reveal that individuals facing food poverty often resort to innovative coping strategies, such as scavenging for food in dumpsters or attending events offering complimentary meals. Some may feel compelled to sacrifice their hobbies or sell their belongings, including online platforms like eBay, to cover the costs of both food and bills. In extreme cases, individuals may consider searching for complimentary food samples online or in natural settings, donating plasma, or even selling their own bodies to secure access to food.

### Multidimensionality of food poverty

The analysis of code relations reveals multifaceted overlaps among subdimensions of food poverty, indicating its multidimensional nature. These overlaps occur within both the material dimension and its corresponding subdimensions, as well as within the social dimensions and their corresponding subdimensions. Notably, significant overlaps exist between the material and social subdimensions (see Figure S1 in the supplementary material). When clustering based on the distance matrix, a single large cluster emerges, encompassing almost all codes, except for ‘coping strategies’ and ‘no facilities’, which show limited overlaps with other food poverty dimensions (see Figure S2 in the supplementary material).

We emphasize overlaps exceeding a cumulative column sum of 50 in our presentation of results. The most overlapping subdimension is the physiological subdimension ‘strategies’ for achieving adequate nutrition with limited financial resources. This subdimension frequently overlaps with the material dimension, especially with economic aspects like policy directives, price increases, and the mid-month phenomenon. It also correlates with other physiological subdimensions, such as the challenge of maintaining a healthy diet under impoverished conditions. Moreover, the ‘strategies’ subdimension significantly overlaps with social subdimensions, notably in the context of social role perceptions, social network maintenance, and symbolic food practices. These findings suggest that physiologically oriented dietary strategies not only impact physical health but also connect to social deprivations within food poverty, influencing role perceptions, social relationships, and cultural practices.

Similar patterns emerge, particularly in the ‘social participation’ subdimension, where the trade-off between food and financing other necessities closely relates to social network maintenance and the perception of social roles. Additionally, ‘social participation’ intertwines with the perception of symbolic food and cultural practices. This interplay results in perceived threats to dignity, leading to justifications among individuals affected by poverty. Conversely, assaults on dignity and associated justifications closely align with feelings of shame and perceived biases against impoverished individuals. Furthermore, these dignity-related aspects are strongly associated with policy demands, particularly regarding increased food assistance and acknowledgment of nutrition as a human right and a matter of dignity.

Furthermore, strong associations are observed in the perception of social roles and dietary strategies, closely linked to emotional states, thoughts, and fears. Similarly, discussions of price increases, inflation, and the challenges of maintaining a healthy diet under poverty exhibit robust associations. This subcategory also significantly correlates with diseases and comorbidities, highlighting the dual burden faced by individuals simultaneously dealing with illness and poverty. Notably, statements about the impossibility of a healthy diet strongly connect with dignity and justifications, revealing societal attitudes toward the dietary practices of impoverished individuals. Overall, it becomes apparent that the individual subdimensions of food poverty exhibit significant interconnections in various configurations, representing a kind of interdependent structure and causal chain. Phenomena observed within one subdimension manifest themselves in other subdimensions with differing attributions of significance.

#IamPovertyAffected and hungry. Hungry for life. Hungry for social contact. Hungry to be a part of society. Hungry for hobbies and new experiences. But I am also really hungry. Because even for regular meals there is no longer enough without help.

As exemplified by this tweet using the term ‘hunger’, food poverty manifests itself in both the material dimensions, such as physiological hunger, and is equally significant in the social dimensions that are impaired by material food poverty.

## Discussion

The study aimed to explore individuals’ subjective experiences of food poverty through the analysis of the Twitter discourse employing the German-language hashtag #IchBinArmutsbetroffen. It sought to uncover nuanced dimensions of food poverty that extend beyond statistical data and theoretical frameworks, offering deeper insights into the multifaceted deprivations faced by affected individuals in their daily lives. The study revealed that food poverty manifests diversely within both the theoretically derived subdimensions of food poverty, namely the material and social dimensions, and becomes a subject of discourse that provides more concrete manifestations within these subdimensions, thereby enriching theoretical discussions. Moreover, the study demonstrated the intricate interconnections between these subdimensions within themselves and across dimensions, resulting in the emergence of multidimensional manifestations of food poverty.

Specifically focusing on the economic subdimension of food poverty, the study’s findings underscored its substantial representation in Twitter discussions, highlighting the inadequacy of financial resources for accessing food. These financial constraints, essential in statistical assessments of food insecurity, often lead to one-dimensional conclusions about dietary behaviours in poverty, emphasizing disparities in dietary practices among individuals of varying economic statuses [8, 64]. However, the discussions within the economic dimension unveiled the intricate dynamics driven by constrained financial means, shedding light on how such constraints impact various aspects of food procurement and daily life. This leads to decisions and economic heuristics involving trade-offs between food and other essential needs, financial sacrifices, and challenges in accessing vital medical expenses for individuals grappling with food poverty. The study aligns with previous observations that food poverty significantly influences individuals’ decision-making processes and food acquisition strategies while inducing emotional distress and impacting social interactions and ‘alimentary participation’ stemming from persistent fears of food scarcity [11, 22].

Furthermore, discussions within the economic subdimension primarily revolved around calls for government intervention, criticism of political inaction, and concerns regarding the adequacy of proposed government assistance, especially considering rising costs. Users of the hashtag strategically employed it not only to convey information and raise awareness about their situation but also to integrate their tweets into broader discussions on food poverty and social policy [38, 41]. This collaborative shaping of information aims to increase visibility, particularly among policymakers, which resonates with the findings of previous research utilizing Twitter data to highlight societal trends related to food poverty [2, 42].

In the physiological subdimension, the study revealed that individuals facing food poverty employ various strategies to attain adequate nutrition despite limited financial resources. These strategies often prioritize hunger alleviation over personal preferences or health considerations. Such strategies are closely associated with the economic dimension and offer insights into the persistent identification of less healthy dietary habits among individuals with low socioeconomic status in nutrition research, despite educational efforts [65, 66]. Additionally, the study highlighted the increased financial burdens and health issues faced by impoverished individuals with specific dietary needs and health challenges. Malnutrition was directly attributed to dietary practices stemming from poverty, shedding light on a previously underexplored aspect of food poverty research.

Conversely, the physical subdimension of food poverty was notably underrepresented, both in German poverty and nutrition research and in the analysed tweets [8]. Key topics included issues such as insufficient food supply from rural food banks and expressions of gratitude when individuals had access to gardens for food production. Some tweets also addressed concerns related to altered or extreme climatic conditions, particularly the potential health impacts of extreme heat. Given innovative studies that utilize Twitter data to identify geographical dynamics or food deserts associated with inadequate access to healthy food [54, 55] and the growing need for research to focus on charity food supply options in rural areas these aspects merit further investigation. Additionally, the risks posed by the climate crisis, particularly for socioeconomically disadvantaged groups, should be integrated more extensively into public health research.

The social subdimension of food poverty illuminated how financial constraints often prevent individuals from participating in social activities involving food, echoing well-established findings in theoretical and empirical research that foods serves as a means for social inclusion and exclusion [10, 25]. Sacrificing interests due to economic limitations underscores the significance of social aspects linked to communal meals and discussions over food and drink [12–15]. Additionally, the tweets emphasized the impact of food poverty on social roles, particularly within the parental role, where parents often prioritize their children’s nutritional needs over their own. This manifestation of the social subdimension, primarily affecting mothers who occasionally forgo meals to ensure their children have enough to eat [23, 24], has received limited attention to date. However, it holds the potential to serve as an early indicator of food poverty by specifically examining the dietary practices of mothers, as they often strive to maintain a semblance of normalcy for their children, making food poverty less visible among them.

Within the cultural subdimension, the most prominent subcategory, ‘symbolic food and practices’, revealed that specific foods continue to symbolize status distinctions and reproduce food-based classism and ‘othering’ for individuals facing poverty [15, 33], encompassing healthy foods, sociocultural trends, seasonal practices, and items linked to social participation. This subdimension further highlighted the impact of food poverty on significant life events and celebrations, where individuals often struggle to financially contribute or prepare customary dishes, leading to anxieties and difficult choices, particularly during festive occasions. The emergence of social organizations and initiatives, such as #Bratenpaten, #wishlistengel, or #einesorgeweniger, within the Twitter discourse illustrated how the hashtag #IchBinArmutsbetroffen promotes solidarity and fosters connections among those affected and supporters [41, 44, 45]. These social innovations distinguish themselves from previous charity approaches, such as food banks, by providing anonymous and more needs-oriented support to individuals in poverty, with the specific aim of mitigating social and cultural deprivations associated with food poverty. Observing their dynamics in future research is certainly worthwhile as they represent a new form of delegation of food poverty, addressing an aspect that has been neglected in standard allowances and political discourses.

Within the psychological subdimension, the study found that discussions regarding social food poverty prominently featured attacks on the dignity and related justifications of individuals facing poverty. These discussions underscored the courageous sharing of experiences on Twitter and the importance of averting shame and criticism, notably in the face of judgment from both online and offline sources. The media’s stigmatizing and stereotypical portrayal of those affected by poverty, who are often accused by political circles of being financially incompetent [17, 18] and by nutrition and health research of making poor dietary decisions or lacking competence [9, 64–66], triggers feelings of indignity among those in poverty, compelling them to justify their situation and choices. To date, nutritional and health research has tended to categorize individuals experiencing poverty into a singular group of malnourished individuals, often overlooking their diverse experiences of deprivation in daily life and neglecting the intricate dynamics of representation that come into play when nutritional and health research assigns stigmatizing advise and labels to those who are socioeconomically vulnerable. The study emphasized that it is inappropriate to overlook an individual’s competence and dignity by suggesting simplistic solutions without considering their financial circumstances and the multidimensionality of food poverty with its various factors.

In conclusion, the #IchBinArmutsbetroffen hashtag has provided a valuable platform for individuals to share their experiences of food poverty and challenge stigmatizing attitudes. The analysis has unveiled the multifaceted nature of food poverty and its interconnected dimensions, contributing to a deeper understanding of this complex issue. It has also highlighted the challenges of affording healthy food during periods of poverty and emphasized the need for a nuanced approach that considers the financial constraints individuals face. Additionally, the study has shown how the narratives of those affected by food poverty are incorporated into broader socio-political and nutritional discourses, perpetuating status differences in dietary behaviours and contributing to the stigmatization of those in poverty. Therefore, it is crucial to recognize the dignity and competence of individuals facing food poverty and refrain from proposing simplistic solutions that disregard their financial circumstances and the multidimensionality of the issue.

### Limitations and future research

The study has several limitations. Firstly, social media data analysis is a complex field that often requires interdisciplinary collaboration and mixed methods approaches to fully utilize its potential in food research [34]. Secondly, the exclusive use of the hashtag #IchBinArmutsbetroffen means that our findings may not generalize to social groups not active on Twitter, introducing potential biases and sampling limitations. An analysis of the discourse surrounding food poverty on other social media platforms could also provide insights into how different age groups perceive this phenomenon. For instance, the discourse on TikTok might offer indications of how adolescents in affected families perceive and experience food poverty. Additionally, not all social media users employ hashtags, so posts from individuals affected by food poverty who do not use this specific hashtag are not included in our dataset. Future research could expand its scope to capture posts about food poverty without relying on this particular hashtag. Furthermore, our analysis focused solely on posts in the German language, limiting our insights to this specific context and excluding other linguistic and cultural contexts. Lastly, the identified overlaps between subdimensions of food poverty should not be considered universally applicable, and their quantity should not be the sole basis for assessment, as they provide only partial insights into the intricate interplay among these subdimensions based on the analysed tweets in this study.

## Conclusion

In this study, we have demonstrated that food poverty is a multifaceted phenomenon with interconnected dimensions, yet research and political attention dedicated to this pressing issue in Germany are limited. Employing a theoretical model of food poverty, this mixed-methods study has elucidated how individuals using the Twitter hashtag #IchBinArmutsbetroffen experience food poverty across various dimensions and how these dimensions overlap, forming a complex network of implications. This research complements previous predominantly quantitative investigations of food insecurity and poverty in Germany by providing a qualitative perspective on the experiences of those affected by food poverty, a group that is often challenging to access in social research. To formulate effective preventive measures and health-oriented nutritional policies, a comprehensive representation of both facets of food poverty is imperative, particularly within countries like Germany. The outcomes of our study offer foundational insights for crafting indicators pertinent to social dimensions of food poverty within the examined group [8]. Additionally, the study highlights the potential of Twitter data for food studies research, demonstrating the extraction of nutrition-related insights from a wealth of available data. Future food studies research addressing poverty should not merely view social media data as a research tool but should also leverage the rich data on individual perceptions and revelations to give voice to the subjective perspectives of those affected and emphasize the multidimensionality of this phenomenon within the scientific discourse.

### Electronic supplementary material

Below is the link to the electronic supplementary material.

Supplementary Material 1

## Data Availability

Access to the dataset (shiny website) is provided via link in the manuscript. Access to the processed dataset analyzed during the current study is available from the corresponding author on reasonable request.

## Footnotes

1 https://twitter.com/Finkulasa/status/1524689204520927237.
