# Supplementary material for: Exploring food poverty experiences in the German Twitter-Sphere
Source: BMC Public Health. 2024 May 25;24:1398. doi: 10.1186/s12889-024-18926-8 (PMC11127365; doi:10.1186/s12889-024-18926-8)
Supplement: Supplementary file 1 — Supplementary Material 1 [file 12889_2024_18926_MOESM1_ESM.docx]

# Supplementary material

# **Exploring Food Poverty Experiences in the German Twitter-Sphere**

Tina Bartelmeß^*^, Mirco Schönfeld & Jürgen Pfeffer

Table S1: Coding scheme with example tweets

| **(Sub)dimension** | **Number of codings** | **Share of total codings** | **Description** | **Example tweets (German)** |
| --- | --- | --- | --- | --- |
| **MATERIAL FOOD POVERTY** | | | | |
| **Economical** | **637** | **33,18%** |  | |
| Political call/criticism | 204 | 32,03% | - Calls for government action - Criticism of politicians for their lack of action | @Bundeskanzler Herr @Bundeskanzler, Haben Sie nichts zu #IchBinArmutsbetroffen zu sagen? Wir warten alle gespannt auf eine Reaktion von Ihnen, denn wir können alles nicht mehr. Wir leben nicht, wir vegetieren. Die Inflation **frisst** uns auf und wir bekommen die Kinder nicht mehr **satt**.  @c_lindner Ich möchte hinweisen, dass 14 Millionen Menschen ab 20ten jeden Monats bereits durchaus **hungern**. Ich **esse** oftmals nur 1 **Mahlzeit** am Tag, da ich auch unter #IchBinArmutsbetroffen falle. Wo leben Sie also? Sie verhindern doch alles, was wir benötigen! Deutlich höhere Regelsätze! |
| Basic income, standard allowances, and social security | 131 | 20,57% | - Critique regarding the perceived inadequacy of the proposed increase in government assistance - Reports concerning budget and expenditures | #IchBinArmutsbetroffen Für 2 Tests pro Woche, 6€ bezahlen ist ein Tag weniger oder gar nix **essen**, jede Woche. 9 €-Ticket bedeutet 2 Tage nix **essen**. 70 € Stromvorauszahlung - kann sich jeder ausrechnen. Und alles von 449 € EM-Rente und Grusi. Aber Lindner feiert groß.  #IchBinArmutsbetroffen und bin Wähler / Anhänger einer Ampelpartei, aber was diese Ampel für uns als Steigerung beschlossen hat, **frisst** allein der erhöhte Abschlag beim Strom auf. Die Inflation, gerade bei **Lebensmitteln**, lässt uns immer tiefer in ein unwürdigeres Leben abfallen. |
| Weighing food vs. other | 129 | 20,25% | - Descriptions of the trade-offs between food and other necessities. - Description of how trade-off considerations are ingrained as a heuristic among those affected by poverty | #IchBinArmutsbetroffen bedeutet auch. sich zu überlegen, ob man die letzten 10 Euro für die benötigten Medikamente ausgibt oder lieber **Essen** kauft...  #IchBinArmutsbetroffen hieß für mich heute eine bezahlte Rechnung zurückzuziehen, um die Laktosefreien **Lebensmittel** für meinen Sohn zu kaufen, damit er die nächsten Tage etwas zu **essen** hat.  #IchBinArmutsbetroffen Ich habe jedes Mal ein Problem damit, wenn ich mir neue Unterwäsche kaufe, weil ich daran denke, wieviel **Essen** ich von dem Geld hätte kaufen können. 😢 #Armut |
| Price increases/  inflation | 86 | 13,50% | - Descriptions of the impact of price increases and inflation on the ability to access (healthy) food | #IchBinArmutsbetroffen bedeutet übrigens auch, bei den aktuellen Preisanstiegen, gerade im Bereich der **Lebensmittel**, im Laden die Preise zu sehen und verzweifelt zu versuchen, NICHT heulend zusammenzubrechen, weil man nicht mehr weiß, wie man noch ausreichend **Essen** kaufen soll.  #IchBinArmutsbetroffen Wie kommt ihr klar mit den gestiegenen Preisen? Ich **esse** hauptsächlich **Kartoffeln**, Reis, **Nudeln**. Einmal die Woche warm! |
| Food banks | 41 | 6,44% | - Discussions regarding the role and responsibility of food banks - Descriptions of food quality and quantity from food banks in one's dietary practices | Ein Tropfen auf den heißen Stein. Was ist mit den vielen #IchBinArmutsbetroffen\|en, die keinen Zugang zur **Tafel** haben? **Tafe**ln sind im Übrigen ein Zusatzangebot. Dass alle Menschen in Deutschland genug zu **essen** und zu **trinken** haben, muss der Staat gewährleisten, nicht das Ehrenamt!  Das Schönste daran, als auf Almosen von der #Tafel angewiesen zu sein, ist die mangelnde Planungssicherheit der **Mangelernährung**. Hab gerade von 500g **Bohnen** mehr als die Hälfte weggeschmissen, weil gammlig. Nur gut, dass ich gut **kochen** und improvisieren kann. #IchbinArmutsbetroffen |
| Mid-month | 40 | 6,28% | - Statements on debt and rationing of food from the middle of the month | Viele alte Menschen ziehen sich zurück und haben kaum was zu **essen** das Wort #Schmalhanstage ist wieder da. Das ist so traurig. 😢 #IchBinArmutsbetroffen  #IchBinArmutsbetroffen und weiß dann ab heute nicht mehr was ich **essen** soll. Es sind noch ein paar **Aufbackbrötchen** da (1 Packung), **Schokoaufstrich** und ein bisschen **Milch**. Es ist der 20. Der Monat noch lang... die Hoffnung klein... Es ist zermürbend 😮‍💨 |
| no facilities | 8 | 1,26% | - Statement on lacking necessary utensils and equipment for food storage or preparation | Ich bin überzeugt, dass man sich auch günstig gesund **ernähren** kann und auch nachhaltiger leben. Das Problem sind einmalige Anschaffungskosten, die für #IchBinArmutsbetroffen e kaum oder gar nicht zu stemmen sind. Ein Tiefkühlschrank hat z.B. nicht jeder.  #IchBinArmutsbetroffen Und habe seit Jahren keinen funktionierenden **Backofen**. Ich habe lange drauf gespart. Aber es waren andere existenzielle Dinge wie z.B. **Essen**, Medikamente, Fahrtkosten und Kleidung die vorrangig sind, für die ich das Geld ausgeben musste. |
| **Physiological** | **459** | **23,91%** |  | |
| Strategies | 185 | 40,31% | - statements on strategies to achieve appropriate nutrition with limited financial resources | Drei Arbeiten, oft sogar zeitgleich, und dennoch Hartz IV. **Essen** gibt es dann, wenn das Kind was auf dem Teller lässt, und so läuft das seit einem Jahr. **Obst**/**Gemüse** wird oft durch **Vitamintabletten** ersetzt, weil die günstiger sind. #IchBinArmutsbetroffen  Etwas **essen**, was eigentlich zu roh oder zu verbrannt ist, weil du es dir nicht leisten kannst **Lebensmittel** wegzuwerfen und du keine Alternative hast. #ichbinarmutsbetroffen |
| Impossibility for healthy nutrition | 140 | 30,50% | - statements on the inability to financially access nutritious and sustainable food options | #IchBinArmutsbetroffen #Berlin **#MinisteriumfürErnährung** Gesundes **Essen** ist für Arme kaum bezahlbar, von fair gehandelten, nachhaltigem und/oder Bio-**Essen** ganz zu schweigen. 2,93€pro Tag für ein Kind sind im #ALGII - Satz pro Tag für **Essen** vorgesehen. Foto: @MariaWa85740570 https://t.co/Gio8NYhLKZ  Ab und zu gibt es bei uns auch mal das böse **#Toastbrot**. Eigentlich immer dann, wenn alles andere vergriffen ist. Vom Preis mal abgesehen. Die Bedeutung von „sich von #Luft und #Liebe“ zu **ernähren**, neu entdeckt. 💭 #IchBinArmutsbetroffen https://t.co/g6DBAY9o8B |
| Diseases/ comorbidities | 81 | 17,65% | - testimonials of individuals suffering from diseases, intolerances, or allergies regarding access to specialized diets | #IchBinArmutsbetroffen bedeutet bei den Lebensmittelpreisen nur fertig **Pizza** für 1.50 zu **Essen**. Mit Gallensteinen natürlich super! Gesundes **Essen** ist zu teuer! **Obst** und **Gemüse**? Zu teuer! Das wirkt sich auch auf die Krankheiten aus… |
| Malnutrition | 53 | 11,55% | - Reports from individuals on perceptions and handling of malnutrition | #Wishlist #IchBinArmutsbetroffen Gesunde, vollwertige **Ernährung** ist mit ALG2 fast nicht möglich, es geht nur darum irgendwie **satt** zu werden. Aufgrund von Mangel**Ernährung** habe ich bereits Haarausfall bekommen. Über eine der Obst- oder **Gemüseboxen** würde ich mich riesig freuen.  #IchBinArmutsbetroffen und wurde heute vom Arzt „angemeckert“, weil meine **Vitamin** B 12 Werte so grottig sind. „**Essen** Sie mehr **Fleisch** und **Milchprodukte**!“ 🤡 **Fleisch**? Ich hörte mal davon, aber das ist laaange her. **Milchprodukte**? Ey Doc, wissen Sie was das kostet? |
| **Physical** | **16** | **0,83%** |  | |
| Supply gaps in rural areas | 5 | 31,25% | - Statements on the institutional and infrastructural supply of food | Bräuchte es auch auf Dörfern. Hier gibt es kein Sharing Containern kein teilen von irgendwas Hier gibt es jeden zweiten Tag nur **Hunger** Heute ist wieder so einer Alle die **Lebensmittel** o **Essen** Rezept o so etwas teilen, machen ihn an diesen Tagen größer #IchBinArmutsbetroffen |
| Self-sufficiency resources | 7 | 43,75% | - Testimonials on self-sufficiency options | Wir haben Spätsommer- **Himbeeren** im Garten 😍 Die gibt es jetzt zum **Frühstück**. #IchBinArmutsbetroffen https://t.co/tXSJmBufJ8 |
| Climatic conditions | 4 | 25,00% | - Statements on concerns and possible health consequences related to climatic conditions | Nächste Woche Temperaturen über 37 Grad. Ich habe Angst. Ich habe Angst, dass mein Kreislauf zusammenklappt. Ich habe Angst ins Krankenhaus zu müssen und kosten zu verursachen. Ich habe Angst, dass ich nichts zu **trinken** habe. #IchBinArmutsbetroffen |
| **sub-total** | **1112** | **57,92%** |  | |
| **SOCIAL FOOD POVERTY** | | | | |
| **Social** | **326** | **16,98%** |  | |
| Social participation | 154 | 47,24% | - Statements and narratives referring to deprivation in social participation | Das preiswerte **Backshopcafé** hat nach Wochen ohne Mitarbeiter heute wiedereröffnet. Ich konnte endlich mal wieder einen **Kaffee** **trinken** gehen. Alle anderen Cafés hier nehmen den doppelten Preis, den ich mir nicht leisten kann. Wochenlang kein Plausch. #IchBinArmutsbetroffen  Heute ist wieder einer dieser Tage... Ich sehe diese Menschen in der **Bäckerei**. Im Café. Sie **genießen** ihr Leben. Sitzen zusammen, lachen. Und ich gehe vorbei. Weil ich es nicht kann. Und ich sehe Ihnen beim Leben zu. #ichbinarmutsbetroffen |
| Social networks | 80 | 24,54% | - Statements on social networks, resources, and relationship management | Spontane Einladung zum **Mittagessen** bekommen. Die Hälfte hab ich einpacken lassen🙈🤗. Bin gar nicht mehr gewohnt mittags zu **essen** 😬🤗❤️ #Ichbinarmutsbetroffen  #IchBinArmutsbetroffen bedeutet: Einladungen (unter Vorwand) ausschlagen aus Scham, kein Geschenk kaufen zu können. Selbst die eigenen Kinder nicht einladen, weil die Kosten für mehr **Essen** nicht drin sind. Keine Teilnahme an dem, was Freunde tun incl. kein Verständnis! |
| Social roles | 73 | 22,39% | - Statements about impairments in the perception of social roles | Es gab Tage da brachte ich meinen Sohn nicht zur Kita weil ich kein **Brot** mehr hatte und ich ihn nicht ohne **essen** in den Kindergarten schicken konnte aus Angst die würden mir das Jugendamt schicken. Ich lebe heute noch mit der Angst wieder da zu landen #IchBinArmutsbetroffen  #IchBinArmutsbetroffen und sehr stolz auf mein erstes Kind, das heute eine Ausbildung macht und nicht Armutsbetroffen ist. Es ging dem Kind immer gut, es musste auf nichts verzichten, hat eine Gesunde **Ernährung** erhalten. Dafür habe ich an mir gespart. |
| Social imprint | 19 | 5,83% | - Statements and narratives about how food poverty imprints people socially | #IchBinArmutsbetroffen verfestigt sich von klein auf. Gestern haben Oma und Opa alles bezahlt. Auf Nachfrage, ob das Kind etwas **essen** oder **trinken** will kam immer die Antwort "ich habe keinen **Hunger**, ich **esse** zuhause was" und "wir haben im Auto noch **trinken**". |
| **Cultural** | **93** | **4,84%** |  | |
| Symbolic food and practices | 73 | 78,49% | - Statements about symbolic meaning of food and dishes and cultural practices | Ich **esse** sehr gern **Hackbraten**. Heute hatte ich dabei ein schlechtes Gefühl, und habe nur die halbe Portion geg**esse**n. Damit ich einmal mehr davon **esse**n kann. Das ist nicht normal. Das ist bescheuert. Kacke ist das. #IchBinArmutsbetroffen  Heute gabs Post ❤️ Reis, etwas zu **Trinken**! und ein **Muffinbackblech**... Ich weine. Vor Freude. Ich kann endlich wieder ein bisschen **backen**. Tausend Dank an jeden, der an uns #IchBinArmutsbetroffen e denkt. An jeden Spender. |
| Festive occasions | 20 | 21,51% | - Inability to participate in cultural activities due to financial constraints | Die Preissteigerungen werden zu Weihnachten für viele traurige Augen sorgen, wenn Eltern ihren Kindern die Frage stellen müssen ob sie an Weihnachten ein warmes **Essen** oder ein gewünschtes Geschenk wollen. #IchBinArmutsbetroffen#  #IchBinArmutsbetroffen und letztes Weihnachten gab's dank @sorgeweniger und den **#Bratenpaten** für meine Familie das traditionelle Weihnachts**essen** meiner Kindheit! https://t.co/4xkNkpqe73 |
| **Psychological** | **389** | **20,26%** |  | |
| Dignity and justification | 178 | 45,76% | - Statements about how dignity is perceived and justifications for maintaining dignity | Das schlimme ist, dass die einem verrechnen wollen, dass man doch richtig gut auskommt, wenn man sich von **Kartoffeln**, **Nudeln**, Reis und **Brot** **ernährt**! Aber ohne andere Sachen wie z.B. **Salat**. Ich könnte kotzen! Es geht niemand was an, was ich **esse**! #IchBinArmutsbetroffen |
| Emotions, thoughts, and fears | 88 | 22,62% | - Expression of emotions and statements about thoughts and fears | #IchBinArmutsbetroffen und #MmB und **esse** häufig nur noch einmal am Tag, was mich aber wirklich frustriert, ist mangelnde Empathie mit den Betroffenen und Perspektiven.  Seit #IchBinArmutsbetroffen bin, habe ich ständig **Hunger** und denke an **Essen**. Ich bin nicht am ver**Hunger**n, aber durch die ständige Angst, nicht genug **Nahrung** über den Monat kaufen zu können, sagt mir mein Körper irgendwie ständig, iss schonmal auf **Vorrat**. 🙄🙄 |
| Shame and prejudices | 70 | 17,99% | - Statements on shame and stigma associated with poverty | #IchBinArmutsbetroffen #SmartMob #Köln #Vorurteile Wir werden reduziert auf "die versaufen und verqualmen ihr Geld" Gesunde **Ernährung** angeblich Fehlanzeige, "**Fastfood** " an der Tagesordnung? Bei 5 Euro am Tag, vielleicht mal ein menschlicher Wunsch danach. https://t.co/x9StO84yia |
| Stress/ depression | 28 | 7,20% | - Statements on feelings of stress and depression due to food poverty | Heute ist wieder einer dieser down Tage. Es ist schönes Wetter draußen, eigentlich würde man gerne ein Eis **essen** gehen, die letzte Oktobersonne **genießen**. Ist es drin? Absolut nicht. Also ist man mal wieder deprimiert zuhause. #ichbinarmutsbetroffen |
| Coping strategies | 25 | 6,43% | - Descriptions of bizarre coping strategies | #IchBinArmutsbetroffen bedeutet leider auch Sachen zu verkaufen die man brauch und Hobbys aufzugeben die Mann liebt. Um Rechnung zu zahlen, **Essen** zu kaufen. |
| **sub-total** | **808** | **42,08%** |  | |
|  | | | | |
| **total** | **1920** | **100,00%** |  | |

Table S2: Code-Relations-Browser-Matrix

| **Codesystem** | **Emotions, thoughts, and fears** | **Strategies** | **Social participation** | **Diseases/ comorbidities** | **Impossibility for healthy nutrition** | **Weighing food vs. other** | **Basic income, standard allowances, and social security** | **Shame and prejudices** | **Social roles** | **Dignity and justification** | **Social networks** | **Price increases/ inflation** | **Mid-month** | **Food bank** | **Political call/ criticism** | **Stress/ depression** | **Symbolic food and practices** | **Malnutrition** | **physical** | **Festive occasions** | **No facilities** | **Social imprint** | **Coping Strategies** | **SUM** |
| --- | --- | --- | --- | --- | --- | --- | --- | --- | --- | --- | --- | --- | --- | --- | --- | --- | --- | --- | --- | --- | --- | --- | --- | --- |
| economical > Political call/ criticism | 4 | 5 | 3 | 0 | 2 | 4 | 8 | 1 | 1 | 6 | 0 | 7 | 0 | 3 | 0 | 0 | 0 | 1 | 0 | 0 | 0 | 0 | 0 | 45 |
| economical > Basic income, standard allowances, and social security | 1 | 2 | 2 | 3 | 8 | 4 | 0 | 3 | 2 | 6 | 0 | 12 | 0 | 1 | 8 | 1 | 2 | 0 | 0 | 0 | 1 | 0 | 0 | 56 |
| economical > Weighing food vs. other | 2 | 3 | 8 | 7 | 4 | 0 | 4 | 3 | 3 | 0 | 1 | 2 | 1 | 0 | 4 | 2 | 0 | 1 | 0 | 2 | 0 | 0 | 0 | 47 |
| economical > Price increases/ inflation | 5 | 9 | 1 | 1 | 5 | 2 | 12 | 1 | 2 | 0 | 0 | 0 | 0 | 0 | 7 | 0 | 3 | 0 | 0 | 1 | 1 | 0 | 0 | 50 |
| economical > Food bank | 1 | 5 | 2 | 2 | 4 | 0 | 1 | 1 | 0 | 1 | 1 | 0 | 0 | 0 | 3 | 2 | 0 | 0 | 1 | 0 | 0 | 0 | 0 | 24 |
| economical > Mid-month | 3 | 6 | 1 | 0 | 1 | 1 | 0 | 1 | 1 | 1 | 1 | 0 | 0 | 0 | 0 | 2 | 0 | 1 | 1 | 0 | 0 | 1 | 0 | 21 |
| economical > No facilities | 0 | 0 | 0 | 0 | 1 | 0 | 1 | 0 | 0 | 0 | 0 | 1 | 0 | 0 | 0 | 0 | 0 | 0 | 0 | 0 | 0 | 0 | 0 | 3 |
| physiological > Strategies | 3 | 0 | 3 | 4 | 8 | 3 | 2 | 3 | 15 | 3 | 6 | 9 | 6 | 5 | 5 | 3 | 7 | 4 | 2 | 0 | 0 | 0 | 0 | 91 |
| physiological > Inpossibility for healthy nutrition | 1 | 8 | 0 | 13 | 0 | 4 | 8 | 2 | 3 | 7 | 0 | 5 | 1 | 4 | 2 | 0 | 2 | 2 | 0 | 0 | 1 | 0 | 1 | 64 |
| physiological > Diseases/ comorbidities | 1 | 4 | 3 | 0 | 13 | 7 | 3 | 1 | 1 | 3 | 1 | 1 | 0 | 2 | 0 | 1 | 1 | 4 | 1 | 1 | 0 | 0 | 0 | 48 |
| physiological > Malnutrition | 2 | 4 | 0 | 4 | 2 | 1 | 0 | 0 | 0 | 1 | 0 | 0 | 1 | 0 | 1 | 1 | 2 | 0 | 0 | 0 | 0 | 0 | 0 | 19 |
| social > Social participation | 3 | 3 | 0 | 3 | 0 | 8 | 2 | 2 | 8 | 6 | 7 | 1 | 1 | 2 | 3 | 2 | 6 | 0 | 0 | 1 | 0 | 0 | 1 | 59 |
| social > Social networks | 3 | 6 | 7 | 1 | 0 | 1 | 0 | 2 | 6 | 1 | 0 | 0 | 1 | 1 | 0 | 2 | 1 | 0 | 1 | 1 | 0 | 0 | 0 | 34 |
| social > Social roles | 4 | 15 | 8 | 1 | 3 | 3 | 2 | 1 | 0 | 0 | 6 | 2 | 1 | 0 | 1 | 0 | 0 | 0 | 0 | 2 | 0 | 3 | 0 | 52 |
| social > Social imprint | 0 | 0 | 0 | 0 | 0 | 0 | 0 | 0 | 3 | 0 | 0 | 0 | 1 | 0 | 0 | 1 | 0 | 0 | 0 | 0 | 0 | 0 | 0 | 5 |
| cultural > Symbolic food and practices | 2 | 7 | 6 | 1 | 2 | 0 | 2 | 1 | 0 | 8 | 1 | 3 | 0 | 0 | 0 | 0 | 0 | 2 | 0 | 0 | 0 | 0 | 0 | 35 |
| cultural > Festive occasions | 4 | 0 | 1 | 1 | 0 | 2 | 0 | 0 | 2 | 0 | 1 | 1 | 0 | 0 | 0 | 0 | 0 | 0 | 0 | 0 | 0 | 0 | 0 | 12 |
| psychological > Dignity and justification | 2 | 3 | 6 | 3 | 7 | 0 | 6 | 7 | 0 | 0 | 1 | 0 | 1 | 1 | 6 | 0 | 8 | 1 | 2 | 0 | 0 | 0 | 0 | 54 |
| psychological > Emotions, thoughts, and fears | 0 | 3 | 3 | 1 | 1 | 2 | 1 | 0 | 4 | 2 | 3 | 5 | 3 | 1 | 4 | 1 | 2 | 2 | 4 | 4 | 0 | 0 | 0 | 46 |
| psychological > Shame and prejudices | 0 | 3 | 2 | 1 | 2 | 3 | 3 | 0 | 1 | 7 | 2 | 1 | 1 | 1 | 1 | 0 | 1 | 0 | 0 | 0 | 0 | 0 | 0 | 29 |
| psychological > Stress/ depression | 1 | 3 | 2 | 1 | 0 | 2 | 1 | 0 | 0 | 0 | 2 | 0 | 2 | 2 | 0 | 0 | 0 | 1 | 1 | 0 | 0 | 1 | 0 | 19 |
| psychological > Coping Strategies | 0 | 0 | 1 | 0 | 1 | 0 | 0 | 0 | 0 | 0 | 0 | 0 | 0 | 0 | 0 | 0 | 0 | 0 | 0 | 0 | 0 | 0 | 0 | 2 |
| SUM | 46 | 91 | 59 | 48 | 64 | 47 | 56 | 29 | 52 | 54 | 34 | 50 | 21 | 24 | 45 | 19 | 35 | 19 | 13 | 12 | 3 | 5 | 2 | 828 |


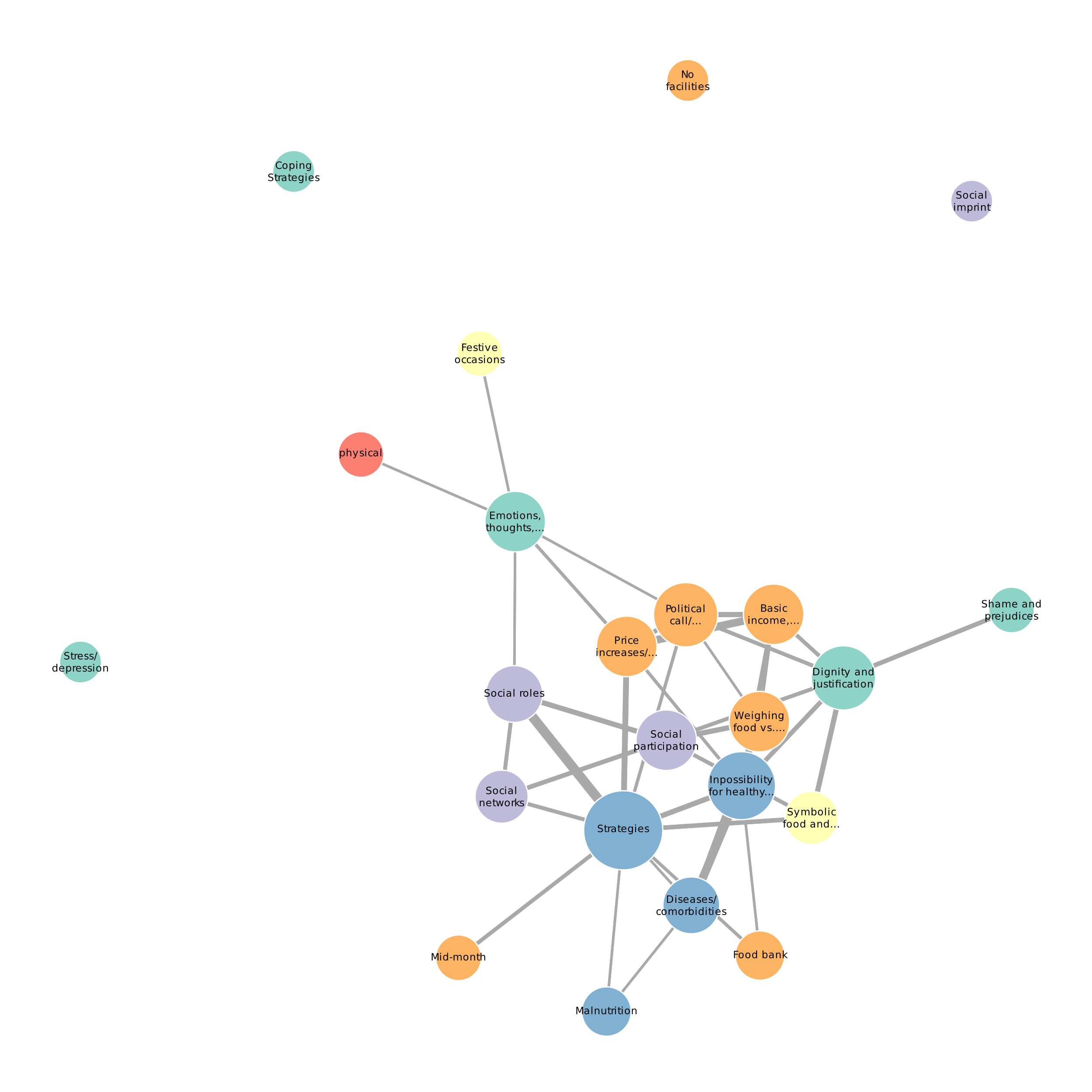


Figure S1: Map of Code-Relations (colours: code system)


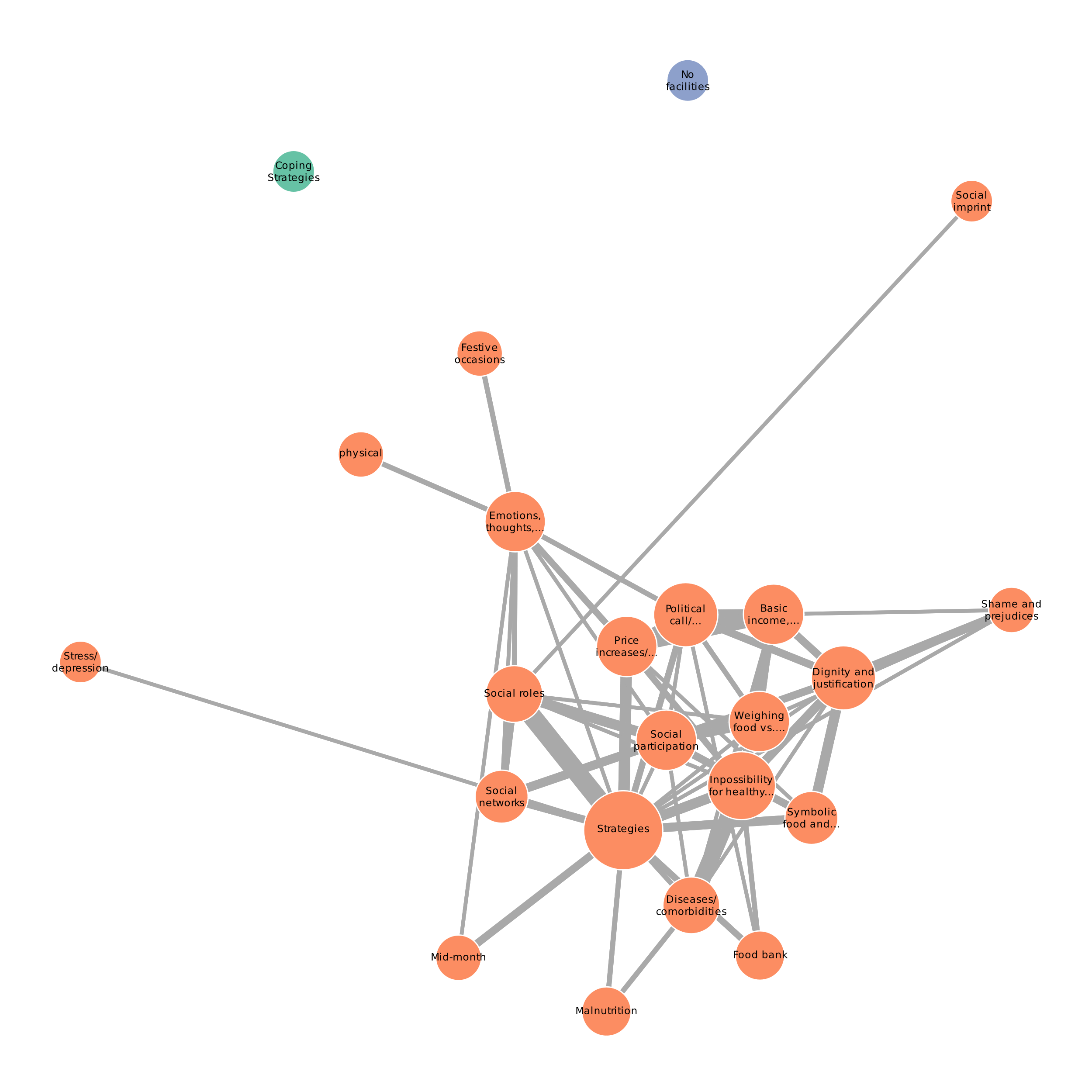


Figure S2: Map of Code-Relations (Colours: distance matrix, 3 clusters)
